# *S*creening the effects of phytoestrogens on lipid metabolism in primary cultured adipocytes from rainbow trout (*Oncorhynchus mykiss*) and gilthead sea bream (*Sparus aurata*)

**DOI:** 10.1007/s10695-025-01483-1

**Published:** 2025-03-25

**Authors:** Sara Balbuena-Pecino, Natàlia Riera-Heredia, Albert Sánchez-Moya, Miquel Perelló-Amorós, Joaquim Gutiérrez, Encarnación Capilla, Isabel Navarro

**Affiliations:** 1 Departament de Biologia Cel·lular, Fisiologia i Immunologia, Facultat de Biologia, Universitat de Barcelona, 08028 Barcelona, Spain; 2 Present Address: Environmental Chemistry Department, IDAEA-CSIC, 08034 Barcelona, Spain

**Keywords:** Genistein, Coumestrol, Estradiol, Phytocompounds, Fish nutrition, Aquaculture

## Abstract

Aquafeed formulation has progressively reduced its dependence on fish-derived ingredients over the past decades. Plant-based substitutes have been a major focus, with soybean meal and its derivatives leading the way. However, many plants contain phytoestrogens, which may affect fish physiology. This study aimed to assess in vitro the effects of genistein (GE), daidzein (DZN), glycitein (GLY), and coumestrol (COU) on the lipid metabolism of rainbow trout (*Oncorhynchus mykiss*) and gilthead sea bream (*Sparus aurata*). Primary cultured adipocytes were incubated with these phytoestrogens, along with 17β-estradiol, at two doses each (1, 10, or 100 μM). The 100 μM dose of GE and DZN decreased adipocyte viability, and mainly enhanced lipid accumulation in both species, suggesting a hypertrophic condition. However, the reduction in adipocyte number and lipid content with 100 μM DZN in rainbow trout indicated a limiting effect on adipose tissue growth in this species. Interestingly, COU significantly increased cell viability in gilthead sea bream, potentially leading to hyperplastic growth, a more favorable metabolic state. In that species, which proved to be more phytoestrogens-sensitive, *lipoprotein lipase* was generally downregulated upon treatments. Moreover, 10 µM GE significantly decreased the mRNA levels of *fatty acid transport protein 1* and *fatty acid synthase*, and increased those of *fatty acid binding protein 1*, suggesting an acceleration of the differentiation process compared to the control cells. This work provides new insights into how dietary phytoestrogens modulate fish lipid metabolism and supports that their presence in plant protein feedstuffs can potentially affect fish health and production performance.

**Supplementary Information:**

The online version contains supplementary material available at 10.1007/s10695-025-01483-1.

## Introduction

Aquaculture is the fastest-growing food-production sector (FAO 2022). Thus, in recent decades there has been an intensification of aquafeeds production, becoming the sustainability of this industry a growing concern (Tacon et al. 2022). In this sense, fish diets have traditionally relied on high concentrations of marine-derived ingredients (i.e., fish meal and fish oil). However, their use has been on a downward trend due to supply and price issues, along with an unsustainable increasing demand from the sector, trying to limit their inclusion in diets to particular production stages (FAO 2022). Consequently, efforts in fish nutrition research have been made to reduce or completely replace these finite resources with alternatives from various other sources, including plants (Hua et al. 2019; Boyd et al. 2020).

Plants synthesize a large number of compounds that can be divided into primary and secondary metabolites. Among the latter, which lack nutritional value but exhibit various biological activities, phytoestrogens are prominent (Nikolić et al. 2017). They are synthetized through different enzymatic pathways, mainly in response to environmental stressors or disease, serving as a protective mechanism (Pavlopoulos et al. 2023). These polyphenolic molecules have a chemical structure very similar to animal estrogens (Dixon 2004; Sirotkin and Harrath 2014). Based on that, phytoestrogens can be classified into flavonoids and non-flavonoids, although most of them belong to the former category (Yildiz 2005). Flavonoids include isoflavones, such as genistein (GE), daidzein (DZN), and glycitein (GLY), whereas non-flavonoids include coumestans, such as coumestrol (COU) (Konar et al. 2012; Nikolić et al. 2017).

Phytoestrogens are biologically active molecules that have the capacity to bind to estrogen receptors (ERs) and function as endocrine disruptors; although they have been described to exhibit not only estrogenic, but also anti-estrogenic activities, both in mammals and fish (Cleveland 2014; Patisaul 2017). The common structural elements essential for their estradiol (E2)-like action include a phenolic ring, necessary for their interaction with the ligand-binding domain of ERs, a low molecular weight similar to E2, a specific distance between two hydroxyl groups in opposite positions, mimicking those of E2, and an optimal hydroxylation pattern (Yildiz 2005).

The steadily increasing incorporation of specific plant-based ingredients, primarily legumes, cereal grains, and oilseeds into commercial fish feeds, with soybean (*Glycine max*) being the most prevalent one among them (Kraugerud et al. 2011; Pavlopoulos et al. 2023), may result in an elevated presence of phytoestrogens in fish tissues, potentially affecting the animal’s physiology (Hardy 2010). In line with this, a recent study by Pavlopoulos et al. (2023) identified and quantified up to 67 phytoestrogens in plant-derived raw materials used for producing fish feeds by means of high-performance liquid chromatography combined with mass spectrometry. The study revealed the presence of 20, 12, and 8 of these phytoestrogens in soybean, sunflower, and rapeseed meal samples, respectively. Moreover, other studies reported the presence of GE and DZN in the blood, liver and muscle tissues of fish fed with commercial feeds containing soybean ingredients (D’Souza et al. 2005; Merlanti et al. 2018; Rzepkowska et al. 2020). In fact, most of the literature on the effects of phytoestrogens in fish has focused on GE, the most abundant one in soybeans (Ng et al. 2006; Chen et al. 2016, 2021; Yang et al. 2022), and their physiological impact on reproductive functions (Bennetau-Pelissero et al. 2001; Green and Kelly 2008; Bagheri et al. 2013). However, less is known about the actions of other phytoestrogens in different tissues, such as adipose tissue, and how they affect metabolism in fish (Balbuena-Pecino et al. 2020).

In terms of lipid metabolism, DZN (5 µg/g body weight), but specially GE (50 µg/g body weight), were found to increase the expression of several genes related to fatty acid synthesis and binding proteins in the liver of rainbow trout (*Oncorhynchus mykiss*) following a 24 h-intraperitoneal injection (Cleveland and Manor 2015). Similarly, in common carp (*Cyprinus carpio*), oral gavage with GE (20 mg/kg body weight) resulted in the upregulation of genes encoding proteins involved in lipid synthesis in the liver, while those associated with lipolysis were downregulated (Yang et al. 2022). In that study, the same pattern was observed in primary cultured hepatocytes incubated with 100 µM GE (Yang et al. 2022). On the other hand, a dietary supplementation with 0.3% GE resulted in a slight increase of the n-3 long chain polyunsaturated fatty acids (LC-PUFA) content in rainbow trout after 8 weeks (Torno et al. 2019), whereas a dose of 15 g/kg dry matter of GE stimulated the biosynthesis of the n-3 docosahexaenoic acid (DHA) in the same species after 52 days of feeding trial (Fickler et al. 2019).

As far as we know, the effects of GLY and COU on fish adipose tissue or lipid metabolism have not been elucidated yet. The interest in understanding the lipogenic process and its regulation stems from the fact that excessive body fat accumulation can lead to negative effects on fish welfare and health, as well as, from a production perspective, affect the quality of the final product (Salmerón et al. 2016). In this context, the present study aimed to characterize the effects of the phytoestrogens GE, DZN, GLY, and COU in comparison to E2 in two important aquaculture commercial species, rainbow trout (*O. mykiss*) and gilthead sea bream (*Sparus aurata*), with a focus on the lipid metabolism of adipose tissue. This screening was conducted using primary cultured preadipocytes from both species, which have been previously demonstrated as a suitable in vitro model for studying the impact of different compounds on adipose tissue physiology in fish (Salmerón et al. 2013; Lutfi et al. 2017).

## Material and methods

### Primary culture of preadipocytes and experimental treatments

Primary cultures of preadipocytes were performed following the previously established protocols described by Bouraoui et al. (2008) for rainbow trout and Salmerón et al. (2013) for gilthead sea bream. Briefly, after mechanical and enzymatic digestion of visceral adipose tissue, the obtained cells were counted and plated on 1% gelatin pre-treated plates. For rainbow trout preadipocytes, cells were plated at a final density of 2–2.5 $\times$ 10^4^ cells/cm^2^ in Leibovitz’s L-15 medium (11415–049, Life Technologies, Alcobendas, Spain) supplemented with 10% fetal bovine serum (F2442, Sigma-Aldrich, Tres-Cantos, Spain) and 1% of antibiotic–antimycotic solution (A5955, Sigma-Aldrich, Tres-Cantos, Spain) (growth medium, GM) at 18ºC. For gilthead sea bream preadipocytes, the seeding concentration was 4.3 $\times$ 10^4^ cells/cm^2^, and the cells were cultured in Dulbecco’s Modified Eagle’s Medium (DMEM)-high glucose (D7777, Sigma-Aldrich, Tres Cantos, Spain) supplemented with 10% fetal bovine serum, 1% antibiotic–antimycotic solution, and 60 mM NaCl (also referred to as GM) at 23ºC with 2.5% CO_2_. The culture medium was changed every 2 days.

Upon reaching confluence (day 7 or 8 of culture for rainbow trout and gilthead sea bream, respectively), cells were induced to differentiate with a differentiation medium (DM), which consisted of GM supplemented with the following components: 10 µg/ml insulin (I6634, Sigma-Aldrich, Tres-Cantos, Spain), 0.5 mM 3-isobutyl-1-methylxanthine (I5879, Sigma-Aldrich, Tres-Cantos, Spain), 0.25 µM dexamethasone (D4902, Sigma-Aldrich, Tres-Cantos, Spain), and 5 μl/ml lipid mixture (L5146, Sigma-Aldrich, Tres Cantos, Spain). At the same time as adding the DM, the phytoestrogens GE (G6649, Sigma-Aldrich, Tres-Cantos, Spain), DZN (D7802, Sigma-Aldrich, Tres-Cantos, Spain), GLY (G2785, Sigma-Aldrich, Tres-Cantos, Spain), COU (11,730, Cayman chemical, Ann Arbor, MI, USA), and E2 (E2758, Sigma-Aldrich, Tres-Cantos, Spain) (used as a positive control of the estrogenic effect) were applied at one or two doses each (1 µM, 10 µM, or 100 µM) for 72 h to determine lipid accumulation, non-esterified fatty acids (NEFA) and glycerol released into the culture medium, and gene expression (Salmerón et al. 2013; Balbuena-Pecino et al. 2020) in both species. To measure cell viability, the same protocol was followed for gilthead sea bream cells. However, in the case of rainbow trout adipocytes, cell viability was evaluated on day 5 after only 24 h, based on a prior study from our group (Balbuena-Pecino et al. 2020); consequently in this case, phytoestrogens were diluted in GM instead of DM. For gene expression analyses, only one dose was used per treatment. Specifically, the highest non-toxic concentration determined by the viability assay was selected, thereby avoiding the use of a dose that could significantly reduce viability and compromise the data.

All phytoestrogens and E2, before being diluted in DM or GM, were first dissolved in DMSO, with the final concentration of the solvent added to the cells, including the control group, being only 0.1%, a dose that has been previously demonstrated to not compromise cell integrity (Lutfi et al. 2017).

### Cell viability assay

The methylthiazolyldiphenyl-tetrazolium bromide (MTT) assay was used to assess cell viability, as previously done for both rainbow trout (Balbuena-Pecino et al. 2020) and gilthead sea bream (Salmerón et al. 2013) primary cultured adipocytes. Briefly, adipocyte samples in duplicate wells from 12-well plates were incubated for the last 18 h of the phytoestrogen treatments with a final concentration of 0.5 mg/ml of MTT (M5655, Sigma-Aldrich, Tres-Cantos, Spain). Then, cells were washed, and the crystals formed were resuspended in DMSO during a 3 h incubation before the spectrophotometric reading. Viability values were obtained from the absorbance measured at 570 nm in duplicate 96-well plates, with the background read at 650 nm subtracted, using a microplate reader (Tecan Infinite M200, Männedorf, Switzerland). Data are presented as fold change relative to the control untreated group (dotted line) (n $=$ 8 independent cell cultures for rainbow trout; n $=$ 6 for gilthead sea bream).

### Cell lipid content determination

Intracellular neutral lipid accumulation was quantified using Oil Red O (ORO) staining, as performed in previous studies using the same in vitro model and species (Balbuena-Pecino et al. 2020; Basto-Silva et al. 2020). After the treatments, adipocyte samples in duplicate wells from 12-well plates were fixed with 10% formalin and stained with 0.3% ORO (O0625, Sigma-Aldrich, Tres-Cantos, Spain). The effectiveness of ORO staining was assessed using a Zeiss Axiovert 40C inverted research-grade microscope. Excess dye was washed off and the stain was resuspended in 2-propanol. Quantification of lipid content was determined by calculating the absorbance ratio at 490 nm to that at 630 nm in duplicate 96-well plates, using a microplate reader (Tecan Infinite M200, Männedorf, Switzerland). The absorbance reading at 630 nm corresponded to the cell protein content, which was measured after staining the cells with Coomassie Brilliant blue G 250 and subsequently extracting it with propylene glycol. Data are presented as fold change relative to the control untreated group (dotted line) (n $=$ 8 independent cell cultures for rainbow trout; n $=$ 6 for gilthead sea bream).

### NEFA and glycerol levels in the culture medium

The levels of NEFA (434–91,795, Wako Chemicals Europe GmbH, Neuss, Germany) and free glycerol (TR0100, Sigma-Aldrich, Tres-Cantos, Spain) released into the culture medium were measured for each experimental condition and cell culture. Quantification was performed using the medium collected from a single well of the 12-well plates used for lipid determination by enzymatic colorimetric methods according to the manufacturers' instructions. Each sample was measured in technical duplicates in a 96-well plate using a microplate reader (Tecan Infinite M200, Männedorf, Switzerland), and NEFA and glycerol concentrations were calculated based on their respective standard curves (n $=$ 8 independent cell cultures for rainbow trout; n $=$ 6 for gilthead sea bream).

### Gene expression analyses

#### RNA extraction and cDNA synthesis

Total RNA was extracted from cell samples collected from two duplicate wells of 6-well plates, which were combined using a cell scraper and 1 ml of TRI Reagent (Applied Biosystems, Alcobendas, Spain), following the manufacturer’s instructions. The quantity of isolated RNA was determined using a NanoDrop 2000 spectrophotometer (Thermo Scientific, Alcobendas, Spain). Afterward, 800 ng or 200 ng of total RNA from rainbow trout or gilthead sea bream, respectively, were treated with DNase I (Invitrogen, Alcobendas, Spain) to remove all genomic DNA, and reverse-transcribed with the Transcriptor First Strand cDNA Synthesis Kit (Roche, Sant Cugat del Vallès, Spain). Finally, the cDNA obtained was stored at $-$ 20 ºC until performing the real-time quantitative PCR (qPCR) analyses.

#### Real-Time Quantitative PCR

The mRNA transcript levels of the target genes plus three reference genes for each species were examined in a CFX384™ Real-Time System (Bio-Rad, El Prat de Llobregat, Spain) following the procedure and qPCR program previously described (Balbuena-Pecino et al. 2019). In brief, reactions were performed in triplicate using 384-well plates with iTaq Universal SYBR Green Supermix (Bio-Rad, El Prat de Llobregat, Spain), 250 nM final concentration of forward and reverse primers (Supplementary Tables S1 and S2 for rainbow trout and gilthead sea bream, respectively), and 1 μl of diluted cDNA sample, in a final volume of 5 μl. The expression level of each gene evaluated was calculated with the Pfaffl method (Pfaffl 2001), relative to the geometric mean of the most stable reference genes. In the case of rainbow trout, these were *elongation factor 1 alpha* (*ef1a*) and *ubiquitin, *and for gilthead sea bream, *ef1α*, *ribosomal protein l27a* (*rpl27a*), and *ribosomal protein s18* (*rps18*). Reference gene stability and relative expression of the target genes were determined using the Bio-Rad CFX Manager Software v. 5. These data are presented in Supplementary Tables S3 and S4 (for rainbow trout and gilthead sea bream, respectively). Additionally, for easier visualization, the gene expression results were standardized through standard score normalization (log2) against the geometric mean of the control group and are presented as heat maps (n = 6–7 independent cell cultures for rainbow trout, n = 5 for gilthead sea bream).

### Statistical analysis

Data were analyzed using IBM SPSS Statistics v. 27 (IBM, Armonk, NY, USA) and plotted as box-and-whisker plots with GraphPad Prism v. 8 (GraphPad Software, La Jolla, CA, USA). For the analysis of normalized data (cell viability and lipid accumulation assays), the statistical significance of each treatment compared to the control group was assessed using the non-parametric Mann–Whitney U test, with the Bonferroni correction. The Bonferroni-adjusted *p* value was set at 0.0055 for rainbow trout (9 comparisons) and 0.005 for gilthead sea bream (10 comparisons). With regards to NEFA and glycerol levels in the culture medium and gene expression results, data normality and homoscedasticity were evaluated by Shapiro–Wilk test and Levene's test, respectively. Next, statistical significance between each treatment and the control group was determined using one-way analysis of variance (one-way ANOVA) followed by Dunnett’s *post-hoc* test. When homoscedasticity was not observed, Dunnett’s T3 test was applied. In these cases, statistical differences were considered significant when *p* < 0.05.

## Results

### Phytoestrogens’ effects on cell viability, lipid content, and NEFA and glycerol levels in the culture medium

**Fig. 1:**
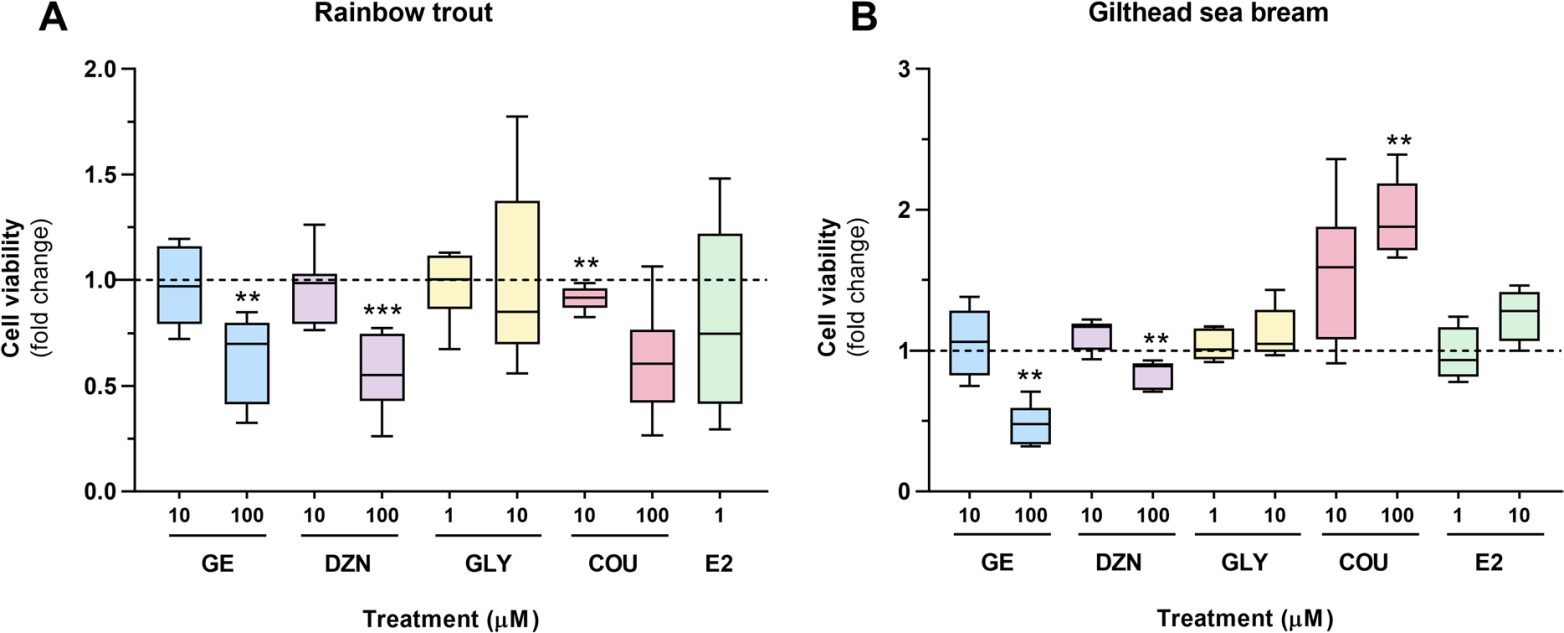
Quantification of cell viability using an MTT assay in (**A**) rainbow trout and (**B**) gilthead sea bream adipocytes incubated on day 5 for 24 h or day 8 for 72 h, respectively, with GE, DZN, GLY, and COU at two doses each (1, 10, or 100 μM), E2 (1 μM, and also 10 μM for gilthead sea bream), or vehicle (GM + 0.1% DMSO for rainbow trout, DM + 0.1% DMSO for gilthead sea bream) as a control. Data are presented as fold change relative to the control condition (dashed line) in box-and-whisker plots (n = 8 independent cell cultures for rainbow trout, n = 6 for gilthead sea bream). Significant differences from the control group were determined by the non-parametric Mann–Whitney U test with Bonferroni correction and are indicated by asterisks. The Bonferroni-adjusted *p*-value was set at 0.0055 for rainbow trout (9 comparisons) and 0.005 for gilthead sea bream (10 comparisons), and shown as **, and when *p* < 0.001 as ***). GE: genistein; DZN: daidzein; GLY: glycitein; COU: coumestrol; E2: 17β-estradiol; GM: growth medium; DM: differentiation medium

In rainbow trout adipocytes, cell viability was significantly reduced by the high dose of GE and DZN (100 µM), as well as by the low dose of COU (10 µM). The remaining groups did not induce significant changes in this parameter compared to the control cells (Fig. 1A). Regarding the adipocytes from gilthead sea bream, cell viability results showed a similar pattern to that observed in rainbow trout, with significantly decreased values after exposure to GE and DZN at 100 µM. However, in this species, a positive dose–response effect was observed after the 72 h-treatment with COU, with the 100 µM concentration causing a significantly higher level of cell viability compared to the control condition without phytoestrogens (Fig. 1B).

**Fig. 2:**
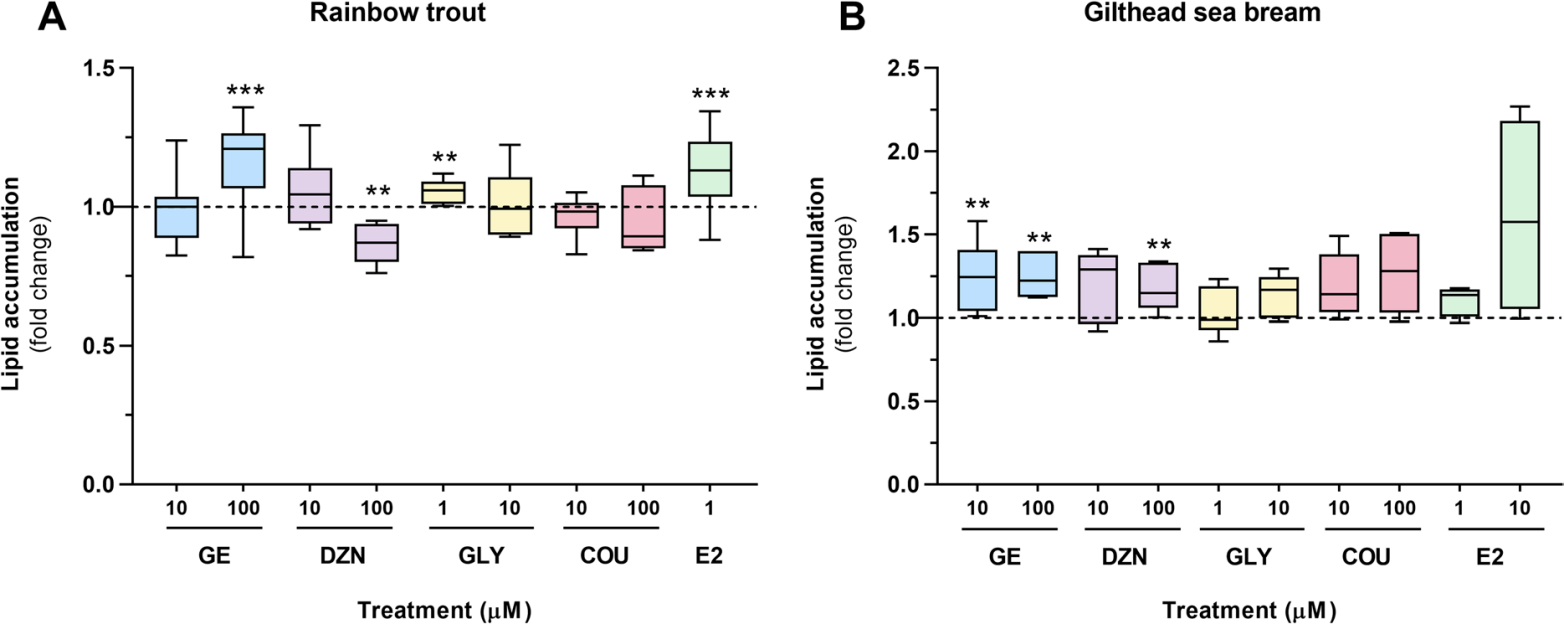
Quantification of lipid content after ORO staining in (**A**) rainbow trout and (**B**) gilthead sea bream adipocytes incubated on day 7 or 8, respectively, for 72 h with GE, DZN, GLY, and COU at two doses each (1, 10, or 100 μM), E2 (1 μM, and also 10 μM for gilthead sea bream), or vehicle (DM + 0.1% DMSO) as a control. Data are presented as fold change relative to the control condition (dashed line) in box-and-whisker plots (n = 8 independent cell cultures for rainbow trout, n = 6 for gilthead sea bream). Significant differences from the control group were determined by the non-parametric Mann–Whitney U test with Bonferroni correction and are indicated by asterisks. The Bonferroni-adjusted *p*-value was set at 0.0055 for rainbow trout (9 comparisons) and 0.005 for gilthead sea bream (10 comparisons), and shown as **, and when *p* < 0.001 as ***). GE: genistein; DZN: daidzein; GLY: glycitein; COU: coumestrol; E2: 17β-estradiol; DM: differentiation medium

Concerning lipid accumulation, the exposure of rainbow trout adipocytes to 100 µM GE, and 1 µM of GLY and E2 significantly increased the lipid content compared to the control group. In contrast, adipocytes treated with the high dose of DZN showed significantly lower lipid levels than the control group (Fig. 2A). Adipocytes from gilthead sea bream exhibited a general trend of increasing this parameter following incubation with the distinct phytoestrogens, although this effect was only statistically significant with both doses of GE (10 and 100 µM) and DZN at 100 µM (Fig. 2B).

**Fig. 3:**
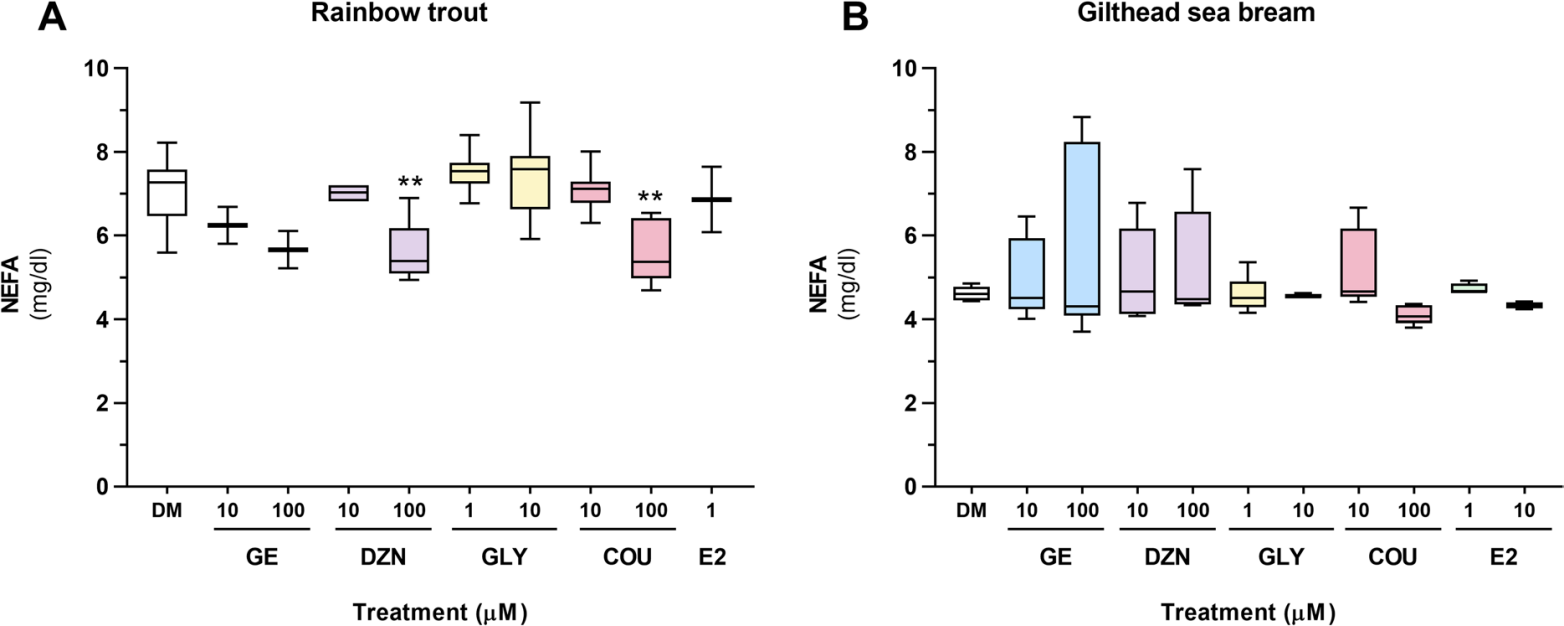
NEFA levels (mg/dl) secreted into the cell culture medium of (**A**) rainbow trout and (**B**) gilthead sea bream adipocytes incubated on day 7 or 8, respectively, for 72 h with GE, DZN, GLY, and COU at two doses each (1, 10, or 100 μM), E2 (1 μM), or vehicle (0.1% DMSO) as a control (DM). Data are presented as box-and-whisker plots (n = 8 independent cell cultures, except for the GE and E2 groups, where n = 2). Significant differences from the control group were determined by one-way ANOVA followed by Dunnett’s *post-hoc* test and are indicated by asterisks (**p < 0.01). GE: genistein; DZN: daidzein; GLY: glycitein; COU: coumestrol; E2: 17β-estradiol; DM: differentiation medium

Regarding the two metabolites measured in the culture medium, NEFA levels in rainbow trout samples were significantly lower in the groups treated with the high dose of DZN and COU compared to the control group (Fig. 3A). In contrast, NEFA levels in gilthead sea bream remained unchanged after incubation with any of the phytoestrogens (Fig. 3B).

**Fig. 4:**
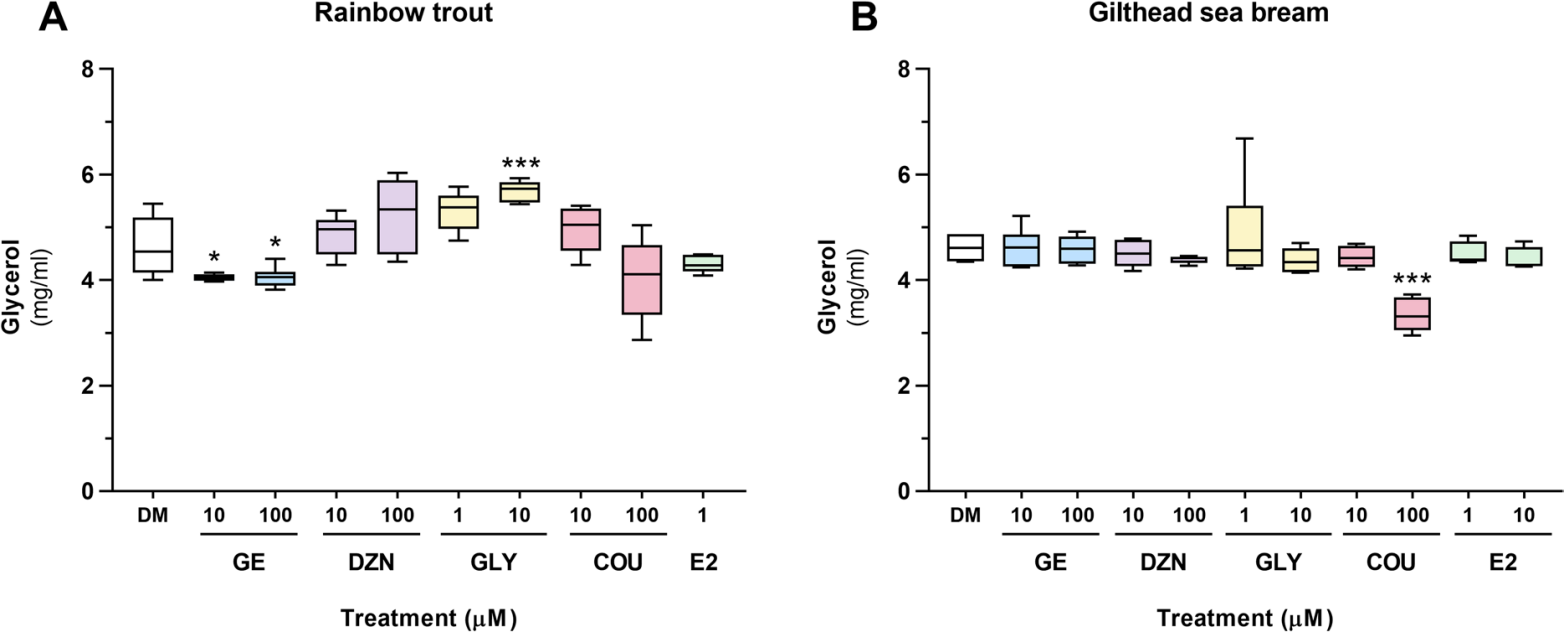
Free glycerol levels (mg/ml) secreted into the cell culture medium of (**A**) rainbow trout and (**B**) gilthead sea bream adipocytes incubated on day 7 or 8, respectively, for 72 h with GE, DZN, GLY, and COU at two doses each (1, 10, or 100 μM), E2 (1 μM), or vehicle (0.1% DMSO) as a control (DM). Data are presented as box-and-whisker plots (n $=$ 6 independent cell cultures). Significant differences from the control group were determined by one-way ANOVA followed by Dunnett’s *post-hoc* test and are indicated by asterisks (*p < 0.05, ***p < 0.001). GE: genistein; DZN: daidzein; GLY: glycitein; COU: coumestrol; E2: 17β-estradiol; DM: differentiation medium

On the other hand, in rainbow trout, glycerol levels were reduced by both doses of GE but were significantly increased after the treatment with GLY at 10 µM (Fig. 4A). In gilthead sea bream, only COU at 100 µM induced a decrease in glycerol levels compared to the control condition (Fig. 4B).

### Phytoestrogens’ effects in adipocytes lipid metabolism-related genes expression

**Fig. 5:**
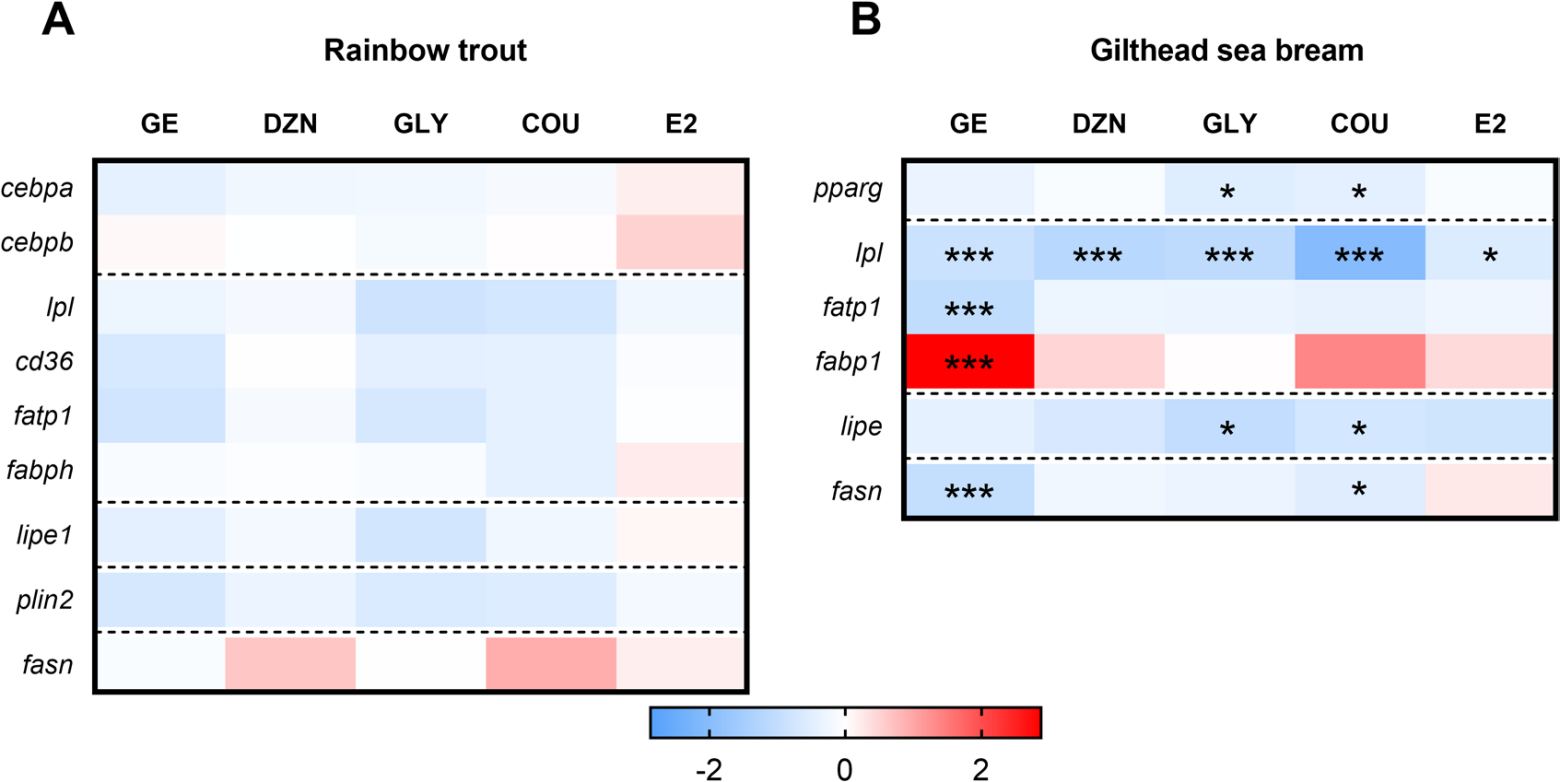
Heat maps comparing the changes in the expression of lipid metabolism-related genes in (**A**) rainbow trout and (**B**) gilthead sea bream adipocytes incubated on day 7 or 8, respectively, for 72 h with GE (10 μM), DZN (10 μM), GLY (10 μM), COU (10 μM for rainbow trout, 100 μM for gilthead sea bream), E2 (1 μM for rainbow trout, 10 μM for gilthead sea bream), or vehicle (DM + 0.1% DMSO) as a control. Relative gene expression data were standardized for the heat map using standard score normalization (log2) against the geometric mean of the control samples (n = 6–7 for rainbow trout, n = 5 for gilthead sea bream). Red and blue shades indicate the highest and lowest expression levels, as shown in the figure’s scale bar. Significant differences from the control group were determined by one-way ANOVA followed by Dunnett’s *post-hoc* test and are indicated by asterisks (*p < 0.05, ***p < 0.001). GE: genistein; DZN: daidzein; GLY: glycitein; COU: coumestrol; E2: 17β-estradiol; DM: differentiation medium

In rainbow trout adipocytes, no significant changes were found in the expression of the lipid metabolism-related genes analyzed after the 72 h-incubation with any of the phytoestrogens tested (Fig. 5A and Supplementary Table S3). Concerning adipocytes from gilthead sea bream, the mRNA levels of *peroxisome proliferator-activated receptor* gamma (*pparg*) and *hormone-sensitive lipase* (*lipe*) were downregulated after exposure to GLY at 10 µM and COU at 100 µM. Furthermore, all tested phytoestrogens, as well as E2, significantly reduced the mRNA levels of *lipoprotein lipase* (*lpl*) compared to the control cells. Finally, treatment with 10 µM GE also in comparison with control adipocytes, significantly upregulated the mRNA levels of *fatty acid binding protein 1* (*fabp1*) and downregulated those of *fatty acid transport protein 1* (*fatp1*) and *fatty acid synthase* (*fasn*). In adipocytes treated with 100 µM COU, the gene expression of *fasn* was also decreased compared to the control condition (Fig. 5B and Supplementary Table S4).

## Discussion

The importance of adipose tissue in fish metabolic homeostasis is beyond question. In this regard, this tissue is no longer considered merely a lipid storage depot; rather, it is recognized as an endocrine organ that participates in the homeostasis of the whole organism (Hue et al. 2023). The effect of phytoestrogens, especially GE, on growth performance or reproductive function has been an active area of research in fish, including the rainbow trout (Bennetau-Pelissero et al. 2001, 2002; Cleveland and Manor 2015). Nonetheless, the full implications of phytoestrogens in fish health, stemming from the increasing dietary incorporation of plant-based ingredients as a sustainable alternative for fish feed production, have not been fully elucidated, especially regarding adipose tissue. Therefore, the present study aimed to assess the impact of three isoflavones (GE, DZN, and GLY) and the coumestan COU on the lipid metabolism of rainbow trout and gilthead sea bream, two major aquaculture species (FAO 2022), through an in vitro approach using primary cultures of preadipocytes.

In terms of cell viability, GE and DZN exhibited a similar effect in both species. Specifically, the 100 µM dose of these phytoestrogens led to a reduced adipocyte viability compared to the control group. This finding aligns with previous studies in human adipocytes, where GE at concentrations of 25 µM or higher reduced cell viability in primary human preadipocytes (Park et al. 2009; Grossini et al. 2018), and in the AML-I human preadipocyte cell line, a marked decline in both cell viability and proliferation was observed at 100 µM or higher after three days in culture (Hirota et al. 2010). Similarly, inhibitory effects on cell proliferation by DZN in a time-dependent manner have also been reported in the 3T3-L1 murine preadipocyte cell line when incubated at concentrations up to 50 µM (He et al. 2016). In various mammalian cancer-derived cell lines from tissues other than adipose, it has been demonstrated that the ability of GE to inhibit cell growth is also dose-dependent (reviewed by Russo et al. 2016). Overall, the data suggest that these two isoflavones may trigger a similar response in both fish and mammals, exhibiting dose-dependent anti-proliferative effects regardless of the cell type. Additionally, in a previous study of our group (Balbuena-Pecino et al. 2020), the observed cell death induced by 100 µM GE in rainbow trout adipocytes was associated mainly with enhanced autophagy than with an apoptotic process, while signs of apoptosis were found in the murine 3T3-L1 cell line (Hwang et al. 2005) and in the AML-I human preadipocytes (Hirota et al. 2010) after exposure to GE (50–200 µM) or both GE and DZN (100 µM) in the latter case. Induction of both autophagic and apoptotic cell death has also been reported upon GE treatment in other cell types, such as several ovarian carcinoma cell lines (i.e., A2780, CaOV3, and ES2), suggesting that these two processes may exist as cooperative or competitive pathways depending on the cellular environment (Gossner et al. 2007).

Besides, in our cells there was an increase in intracellular lipid accumulation in the same experimental groups, except for rainbow trout adipocytes incubated with 100 µM DZN, where the opposite result was observed. Adipose tissue expansion can occur through hypertrophy (enlargement of existing adipocytes), hyperplasia (increment in adipocyte number arising from precursor cells differentiation), or a combination of both (Choe et al. 2016). According to our results, the decrease in cell viability, together with the increase in lipid deposition induced by GE in both species, as well as by DZN in gilthead sea bream, suggests a hypertrophic state of the cells. Unlike rainbow trout, gilthead sea bream appears to be more sensitive to these isoflavones, showing increased lipid deposition in adipocytes even with the lower doses, although 10 µM DZN did not reach statistical significance. In mammals, it has been shown that the manner in which adipose tissue expands and remodels (i.e., hypertrophy or hyperplasia) plays a crucial role in determining whether obesity leads to metabolic disorders (Sakers et al. 2022). In this regard, hypertrophic growth of adipose tissue is a risk factor associated with higher levels of inflammation, fibrosis, and hypoxia, and it also correlates with metabolic dysfunction (Vishvanath and Gupta 2019; Sakers et al. 2022). Some studies have shown that fish may also exhibit similar characteristics of pathological adipose tissue remodeling under specific circumstances. This has been demonstrated in gilthead sea bream fed vegetable oil-substituted diets (Cruz-Garcia et al. 2011), in zebrafish (*Danio rerio*) overfed with a high-fat diet (Landgraf et al. 2017), and more recently in grass carp (*Ctenopharyngodon idellus*) fed with a high-fat diet supplemented with rosiglitazone, a PPARg agonist (Wei et al. 2024).

According to our data, an elevated dietary intake of GE in both species, as well as DZN in gilthead sea bream, may lead to a potential dysregulation of lipid metabolism that could impact the fish health status, especially with sustained exposure, since once adipocytes reach their maximum lipid storage capacity, cell death occurs, triggering inflammation and fibrosis in the tissue (Vishvanath and Gupta 2019). Conversely, the noted decrease in adipocyte viability and lipid accumulation parameters following exposure to the high dose of DZN in rainbow trout might contribute to a long-term reduction in adipose tissue volume from an in vivo perspective. Most studies on mammalian adipocytes exposed to these isoflavones have shown inhibitory effects on adipogenesis modulation (reviewed by Wang et al. 2017), but some reported biphasic effects, depending on the dose (Dang et al. 2003; Dang and Löwik 2004; Penza et al. 2006), also revealing obesogenic properties (reviewed by Xiang et al. 2023). For instance, DZN decreased intracellular fat deposition in 3T3-L1 adipocytes at concentrations of 50, 100, and 200 µM (He et al. 2016), while it increased it at a concentration of 1 µM (Hall et al. 2019), and in AML-I cells at concentrations of 100 and 200 µM (Hirota et al. 2010). These findings suggest that the effects may depend on experimental designs and/or the interaction with specific receptors of the target cells, as evidenced by the differences between the two species evaluated in our study. In any case, comparing our data with previous literature is challenging not only due to the wide variations in treatment conditions and cell models used, but also because of the inclusion or exclusion of differentiation inducers in the culture media during phytoestrogen incubation.

Additionally, the mentioned rise in lipid content in rainbow trout adipocytes exposed to GE was also observed in those cells treated with E2, suggesting that, at least in this species, the impact of GE on lipid accumulation could be mediated through ERs. Nevertheless, GE and DZN modulate adipogenesis and lipid accretion via complex interactions among multiple pathways (e.g., AMPK, PKA, tyrosine kinase, ERK1/2, etc.), in addition to estrogen-mediated signaling (reviewed by Wang et al. 2017). While the mechanisms of action of these phytoestrogens in fish remain to be explored, it has to be taken into account that the amount of fat in the peritoneal cavity (perivisceral fat) not only can affect fish health, but it is also a crucial product quality aspect to consider, since adiposity can adversely affect consumers’ perception due to its visual and olfactory impact, and may even shorten the shelf-life of the edible fraction (Grigorakis 2007).

Apart from intact adipose tissue, isolated adipocytes also secrete significant amounts of glycerol (Rotondo et al. 2017), being released into the medium after the sequential action of three neutral lipases —adipose triglyceride lipase, hormone-sensitive lipase, and monoacylglycerol lipase— that hydrolyze fatty acids from triacylglycerol stores (lipolytic origin) (Turchini et al. 2022). While the measurement of NEFA and glycerol, the end-products of lipolysis (Langin 2006), in cell culture media did not reveal any differences in gilthead sea bream adipocytes after treatment with GE or DZN, the observed increase in lipid accumulation in rainbow trout adipocytes incubated with 100 µM GE is consistent with the lower glycerol levels found in that group’s medium compared to the control. In the case of rainbow trout adipocytes treated with 100 µM DZN, the reduction in their lipid content was not accompanied by the expected higher secretion of NEFA, although it is worth noting that glycerol levels were indeed higher, albeit not statistically significant. This is in agreement with the findings reported by Rotondo et al. (2017), where an increase in lipolysis was found in primary rat mature epidydimal adipocytes after glucose exposure, even though this change was not consistently reflected in the medium NEFA levels, assuming that the majority of free fatty acids generated by intracellular lipolysis were recycled.

Regarding the third isoflavone tested in the present study, GLY had no impact on adipocyte viability in either species, nor on lipid accumulation in gilthead sea bream. Nevertheless, the low dose (1 µM) resulted in significantly increased lipid deposition in rainbow trout adipocytes, while the high dose (10 µM) induced greater glycerol efflux. In the latter case, newly formed glucose-derived glycerol may account for the production of glycerol by adipocytes in the absence of changes in lipid accumulation (glycolytic origin) (Del Mar Romero et al. 2015). To our knowledge, there are limited reports about the biological effects of GLY in fish, since despite being particularly present in soybeans, it is less abundant compared to GE and DZN (6.5 and 5 times less, respectively). Thus, one study evaluated its effects on estrogen metabolism in the liver and kidney of rainbow trout, both individually at a concentration of 1 µM and in a mixture of GE/DZN/GLY (at a ratio of 1.3:1:0.2, respectively, mirroring the natural proportion found in soybeans) (Ng et al. 2006). A second study, investigated the dietary impact of the same isoflavone mixture (1:1:0.144) on the growth, reproduction and health on the same salmonid species (Pastore et al. 2018). Using mammalian models, Choi et al. (2020) found, consistent with our results in gilthead sea bream, that the viability and lipid accumulation of 3T3-L1 preadipocytes were not affected after a 48-h treatment with GLY at concentrations ranging from 25 to 100 µM. However, in other studies conducted on the same cells but with a longer exposure period (i.e., 10 days), GLY exhibited a fat inhibitory capacity at concentrations of 1 µM (Kim et al. 2016) and 50 µM (Kwon et al. 2006). Therefore, it cannot be ruled out that under the same in vitro experimental conditions, but with prolonged treatment, or even in vivo through the sustained administration of feeds containing ingredients rich in GLY, such as soybean meal, this phytoestrogen could have significantly greater effects in the fish species investigated in the present study.

With COU, the pattern observed in terms of cell viability appeared to be dose-dependent, similar to what was noticed with GE and DZN. However, COU also exhibited species-specific differences, reducing viability in rainbow trout but having the opposite effect in gilthead sea bream, suggesting a potential increase in the number of adipocytes in the latter species due to induced cell proliferation. This compound has been reported to stimulate the viability of rat bone marrow stromal cells at concentrations of 0.01 and 0.1 µM from 24 to 72 h in a time-dependent manner, whereas higher doses (i.e., 1 and 10 µM) repressed it (Wu et al. 2009). Additionally, cell viability was not affected in other in vitro studies with 3T3-L1 adipocytes exposed to COU at concentrations of 10, 50 or 100 µM for 48 h (Li et al. 2015) or at 20, 40 or 60 µM for 24 h (Jang et al. 2016). However, in a previous in vivo study by Kim et al. (2020), the mitotic index of brown adipocytes in mice fed a high-fat diet and treated with COU for 2 weeks was also found to increase, pointing that those cells underwent proliferation and cellular expansion, similar to the findings of the present study. With regards to lipid accumulation, no significant changes were detected in either species, although the values in gilthead sea bream showed a tendency to be higher in response to COU. These results may also support a hyperplastic condition in that species, characterized by the presence of smaller and more numerous adipocytes, which is known to be more metabolically favorable (Sakers et al. 2022). Based on mammalian literature, coumestans have the most pronounced estrogenic effect of all phytoestrogens (Nikolić et al. 2017), although they also exert metabolic effects regardless of their estrogenic properties (Dixon 2004). Unfortunately our study does not provide conclusive results regarding the possible ERs involved in COU actions, highlighting the need for further investigation into this phytoestrogen.

Concerning the transcriptional profile of lipid metabolism markers in rainbow trout adipocytes following the 72-h treatment with the different phytoestrogens, no significant changes were observed compared to the control group. In contrast, gilthead sea bream showed a general pattern of downregulation for most of the genes. Analyzing in detail the gene expression regulation in this species, after 72 h of treatment with GLY and COU (10 µM and 100 µM, respectively), the mRNA levels of the key transcription factor *pparg* were significantly lower compared to the control group. Indeed, species-specific differences have been reported regarding its expression pattern, both at the transcript and protein levels, throughout adipogenesis (reviewed by Salmerón, 2018). However, *pparg* is recognized in any case as the master regulator of the adipogenic program, coordinating the expression of adipocyte-specific genes (Rutkowski et al. 2015). Therefore, the current results agree with its mRNA levels peaking prior to the onset of differentiation, followed by a subsequent decrease, as previously observed in the same species (Salmerón et al. 2016), and suggest that the action of those two phytoestrogens may accelerate this process. On the other hand, the mRNA levels of *pparg* in the rainbow trout samples were almost undetectable (data not shown), consistent with the marked decrease in its expression in primary adipocyte cells of this species from day 9 onward during normal culture development (Riera-Heredia et al. 2022).

Moreover, compared to the control group, significant downregulation of the early marker *lpl* was observed after incubation with the different phytoestrogens and E2 in gilthead sea bream. Once differentiation is committed, the mRNA levels of *lpl* have been demonstrated to decrease in gilthead sea bream adipocytes (Salmerón et al. 2016), mirroring what occurs in the early differentiation stages of adipocytes from rainbow trout (Riera-Heredia et al. 2022) and Atlantic salmon (*Salmo salar*) (Todorčević et al. 2010). Regarding fatty acid uptake and intracellular transport-related genes, in cells treated with 10 µM GE, *fatp1* was decreased while *fabp1* was increased. In addition, and in contrast to rainbow trout, *cd36* mRNA levels were not detected in this species under the current conditions. Huang et al. (2010) proposed that *fatp1* may play a role in initiating preadipocyte differentiation, whereas higher levels of other *fabp* were observed in mature adipocytes (Todorčević et al. 2008; Huang et al. 2010). Overall, it can be hypothesized that these phytoestrogens, especially GE, accelerate the adipocyte differentiation process in gilthead sea bream cells compared to the control condition by enhancing lipid provision. Besides, the transcript levels of the lipolytic lipase *lipe*, which were transiently downregulated during differentiation in adipocytes from the same species (Salmerón et al. 2016), were generally reduced with the phytoestrogens; however, this reduction was only significant in the GLY and COU groups, suggesting that lipolysis is decreased with these treatments, although this was not reflected in the glycerol levels measured in the culture medium. Finally, the process of de novo lipogenesis, which is the synthesis of fatty acids from non-lipid precursors (Proença et al. 2014), was less stimulated in adipocytes exposed to GE and COU compared to control cells, as mRNA levels of *fasn*, an important marker of this pathway (Turchini et al. 2022), were lower. Although this process is known to play a minor role in the production of lipids for storage in fish adipocytes (accounting for less than 0.1% of the total) (Bou et al. 2016), it is usually even decreased in obese animals or those with insulin resistance (Song et al. 2018). This reduction aligns with the higher lipid content observed in the cells exposed to GE and COU in our study (though not statistically significant for the latter), as well as the hypertrophic phenotype of GE-incubated adipocytes. In fact, in mammals, the decreased expression of de novo lipogenesis-related genes appears to contribute to the development of insulin resistance (Song et al. 2018).

Overall, from an applied point of view, although the total intake of phytoestrogens relies on feed consumption and diet composition, the fish ingestion of GE and DZN may range from 1 to 13 µg/g of body weight per day (based on feed intake levels ranging from 0.5% to 4% of body weight) in a hypothetical diet where half of the protein comes from soybean meal and soy protein concentrate (Cleveland and Manor 2015). Moreover, it has been observed in soybean that the individual content of these isoflavones can also vary substantially due to differences in crop variety, location and year (Wang et al. 2000). In previous studies, among the commonly used raw materials or ingredients for fish feeds, all the phytoestrogens evaluated in this study were present in soybean meal samples (Hutabarat et al. 2000; Pavlopoulos et al. 2023), while only traces of GE, DZN and COU were found in rapeseed or sunflower meal samples, and none of them were detected in wheat meal samples (Pavlopoulos et al. 2023). With regards to their presence in commercial oils, scarce amounts of all of them were determined in crude soybean oil (Dou et al. 2020; Pavlopoulos et al. 2023). To date, there are no established upper limits recommended for phytoestrogens in fish feed (Johny 2020). However, it seems to be challenging, since their effects vary among fish species, as demonstrated in this study. In any case, the current data provide new information on the characterization of their effects on lipid metabolism, which is relevant for assessing its potential health risks for fish.

Thus, these findings highlight the need to consider the amount of phytoestrogens, especially GE and COU, in fish diets, particularly in feeds with a high percentage of plant-based ingredients. This consideration should be specific to each species, as they may exhibit different sensitivity to the phytoestrogens, to ultimately improve the sustainability of aquaculture feed production without compromising the health and lipid metabolism of the animals.

## Supplementary Information

Below is the link to the electronic supplementary material.

Supplementary file1 (DOCX 33.5 KB)

Supplementary file2 (DOCX 39.1 KB)

## Data Availability

No datasets were generated or analysed during the current study.
