# Supplementary material for: Screening the effects of phytoestrogens on lipid metabolism in primary cultured adipocytes from rainbow trout (Oncorhynchus mykiss) and gilthead sea bream (Sparus aurata)
Source: Fish Physiol Biochem. 2025 Mar 25;51(2):71. doi: 10.1007/s10695-025-01483-1 (PMC11937063; doi:10.1007/s10695-025-01483-1)
Supplement: Supplementary file 1 — Supplementary file1 (DOCX 33.5 KB) [file 10695_2025_1483_MOESM1_ESM.docx]

**Table S1.** Primers used in the Real-Time quantitative PCR analyses for rainbow trout.

| **Type** | **Gene** | **Primer sequences (5’–3’)** | **Ta (ºC)** | **Accession number** |
| --- | --- | --- | --- | --- |
| **Reference** | *ef1a* | **F**: TCCTCTTGGTCGTTTCGCTG  **R**: ACCCGAGGGACATCCTGTG | 58 | NM_001124339 |
|  | *ubiquitin* | **F**: ACAACATCCAGAAAGAGTCCA  **R**: AGGCGAGCGTAGCACTTG | 58 | NM_001124194 |
| **Lipid metabolism** | *cebpa* | **F**: TGTGGCGATAAAGCAAGAGC  **R**: CTGGTGGGAATGGTGGTAGG | 57 | DQ423469 |
|  | *cebpb* | **F**: CACAAAGTGCTGGAACTGGC  **R**: TGGCACAGCGATAAATGGGT | 60 | NM_001124447 |
|  | *lpl* | **F**: TAATTGGCTGCAGAAAACAC  **R**: CGTCAGCAAACTCAAAGGT | 59 | AJ224693 |
|  | *cd36* | **F**: CAAGTCAGCGACAAACCAGA  **R**: ACTTCTGAGCCTCCACAGGA | 62 | AY606034 |
|  | *fatp1* | **F**: AGGAGAGAACGTCTCCACCA  **R**: CGCATCACAGTCAAATGTCC | 60 | XM_036941441 |
|  | *fabph* | **F**: CGACAGAAAAACAATGACCGTT  **R**: TATGTCCTCACCGCAACCAC | 54 | NM_001165105 |
|  | *lipe1* | **F**: AGGGTCATGGTCATCGTCTC  **R**: CTTGACGGAGGGACAGCTAC | 58 | NM_001197209 |
|  | *plin2* | **F**: GATGGCAATGAGGCAGAGAACA **R**: AGGCAGAGTGGCTAAGGGACAG | 60 | XM_021578132.2 |
|  | *fasn* | **F**: TGCTGGGCTGAACATGACCT  **R**: AGACCGTACAGACCTGGCTTC | 57 | XM_036960735 |

F: forward; R: reverse; Ta: annealing temperature; *ef1α*: elongation factor 1-alpha; *cebpa*: CCAAT/enhancer binding protein alpha; *cebpb*: CCAAT/enhancer binding protein beta; *lipe1*: hormone-sensitive lipase 1; *lpl*: lipoprotein lipase; *cd36*: cluster of differentiation 36; *fatp1*: long-chain fatty acid transport protein 1; *fabbph*: fatty acid binding protein, heart; *plin2*: perilipin-2; *fasn*: fatty acid synthase.

**Table S2.** Primers used in the Real-Time quantitative PCR analyses for gilthead sea bream.

| **Type** | **Gene** | **Primer sequences (5’–3’)** | **Ta (ºC)** | **Accession number** |
| --- | --- | --- | --- | --- |
| **Reference** | *ef1a* | **F**: CTTCAACGCTCAGGTCATCAT  **R**: CACAGCGAAACGACCAAGGGGA | 60 | AF184170 |
|  | *rps18* | **F**: GGGTGTTGGCAGACGTTAC  **R**: CTTCTGCCTGTTGAGGAACCA | 60 | AM490061 |
|  | *rpl27a* | **F**: AAGAGGAACACAACTCACTGCCCCAC  **R**: GCTTGCCTTTGCCCAGAACTTTGTAG | 68 | AY188520 |
| **Lipid metabolism** | *pparg* | **F**: CGCCGTGGACCTGTCAGAGC  **R**: GGAATGGATGGAGGAGGAGGAGATGG | 66 | AY590304 |
|  | *lpl* | **F**: GAGCACGCAGACAACCAGAA  **R**: GGGGTAGATGTCGATGTCGC | 60 | AY495672 |
|  | *cd36* | **F**: CCTTGAGGTTTGCCAAGAGGA  **R**: ATCCAACGCAGCCGTCTCA | 60 | XM_030440140 |
|  | *fatp1* | **F**: CAACAGAGGTGGAGGGCATT  **R**: GGGGAGATACGCAGGAACAC | 60 | XM_030407649 |
|  | *fabp1* | **F**: TTCTCACTTTACCATTGCGGC  **R**: AGTTCATCAGGGAGACCAATCG | 60 | XM_030418150 |
|  | *lipe* | **F**: GCTTTGCTTCAGTTTACCACCATTTC  **R**: GATGTAGCGACCCTTCTGGATGATGTG | 60 | XM_030393851 |
|  | *fasn* | **F**: TGGCAGCATACACACAGACC  **R**: CACACAGGGCTTCAGTTTCA | 60 | XM_030399576 |

F: forward; R: reverse; Ta: annealing temperature; *ef1α*: elongation factor 1-alpha; *rps18:* 40S ribosomal protein S18 *rpl27a:* ribosomal protein L27a; *pparg:* peroxisome proliferator activated receptor gamma; *lipe:* lipase E, hormone-sensitive lipase; *lpl:* lipoprotein lipase; *cd36:* cluster of differentiation 36; *fatp1*: long-chain fatty acid transport protein 1; *fabp1*: fatty acid binding protein 1; *fasn*: fatty acid synthase.
