# Supplementary material for: Screening the effects of phytoestrogens on lipid metabolism in primary cultured adipocytes from rainbow trout (Oncorhynchus mykiss) and gilthead sea bream (Sparus aurata)
Source: Fish Physiol Biochem. 2025 Mar 25;51(2):71. doi: 10.1007/s10695-025-01483-1 (PMC11937063; doi:10.1007/s10695-025-01483-1)
Supplement: Supplementary file 2 — Supplementary file2 (DOCX 39.1 KB) [file 10695_2025_1483_MOESM2_ESM.docx]

**Table S3.** Relative gene expression of lipid metabolism-related genes of rainbow trout adipocytes incubated at day 7 for 72 h with GE (10 μM), DZN (10 μM), GLY (10 μM), COU (10 μM), E2 (1 μM) or vehicle (DM + 0.1% DMSO) as control.

|  | **Control** | **GE 10 μM** | **DZN 10 μM** | **GLY 10 μM** | **COU 10 μM** | **E2 1 μM** |
| --- | --- | --- | --- | --- | --- | --- |
| *cebpa* | 0.793 ± 0.17 | 0.582 ± 0.07 | 0.668 ± 0.04 | 0.675 ± 0.13 | 0.713 ± 0.16 | 0.904 ± 0.15 |
| *cebpb* | 0.487 ± 0.14 | 0.511 ± 0.06 | 0.477 ± 0.03 | 0.433 ± 0.04 | 0.492 ± 0.08 | 0.687 ± 0.09 |
| *lpl* | 0.956 ± 0.21 | 0.773 ± 0.25 | 0.843 ± 0.13 | 0.534 ± 0.09 | 0.569 ± 0.08 | 0.805 ± 0.10 |
| *cd36* | 0.934 ± 0.16 | 0.578 ± 0.07 | 0.939 ± 0.09 | 0.675 ± 0.14 | 0.686 ± 0.09 | 0.877 ± 0.18 |
| *fatp1* | 0.972 ± 0.22 | 0.566 ± 0.12 | 0.870 ± 0.10 | 0.588 ± 0.07 | 0.713 ± 0.09 | 0.941 ± 0.13 |
| *fabph* | 0.742 ± 0.10 | 0.677 ± 0.17 | 0.701 ± 0.14 | 0.681 ± 0.15 | 0.544 ± 0.08 | 0.861 ± 0.10 |
| *lipe1* | 1.054 ± 0.18 | 0.766 ± 0.15 | 0.915 ± 0.10 | 0.616 ± 0.09 | 0.878 ± 0.18 | 1.125 ± 0.13 |
| *plin2* | 1.015 ± 0.30 | 0.617 ± 0.14 | 0.807 ± 0.06 | 0.659 ± 0.09 | 0.679 ± 0.11 | 0.886 ± 0.10 |
| *fasn* | 0.337 ± 0.13 | 0.310 ± 0.11 | 0.524 ± 0.07 | 0.329 ± 0.10 | 0.635 ± 0.14 | 0.384 ± 0.11 |

Data are shown as mean ± SEM (n=6-7). Significant differences with the control group were determined by one-way ANOVA, followed by Dunnett’s *post-hoc* test. DM: differentiation medium; GE: genistein; COU: coumestrol; DZN: daidzein; GLY: glycitein; COU: coumestrol; E2: 17β-estradiol.

**Table S4.** Relative gene expression of lipid metabolism-related genes of gilthead sea bream adipocytes incubated at day 8 for 72 h with GE (10 μM), DZN (10 μM), GLY (10 μM), COU (100 μM), E2 (10 μM) or vehicle (DM + 0.1% DMSO) as control.

|  | **Control** | **GE 10 μM** | **DZN 10 μM** | **GLY 10 μM** | **COU 100 μM** | **E2 10 μM** |
| --- | --- | --- | --- | --- | --- | --- |
| *pparg* | 1.218 ± 0.08 | 0.945 ± 0.04 | 1.131 ± 0.11 | 0.836 ± 0.07^*^ | 0.882 ± 0.03^*^ | 1.133 ± 0.12 |
| *lpl* | 1.054 ± 0.18 | 0.766 ± 0.15^***^ | 0.915 ± 0.10^***^ | 0.616 ± 0.09^***^ | 0.878 ± 0.18^***^ | 1.125 ± 0.13^*^ |
| *fatp1* | 1.047 ± 0.08 | 0.504 ± 0.03^***^ | 0.844 ± 0.10 | 0.833 ± 0.06 | 0.793 ± 0.07 | 0.859 ± 0.10 |
| *fabp1* | 0.019 ± 0.007 | 0.137 ± 0.03^***^ | 0.026 ± 0.008 | 0.019 ± 0.001 | 0.048 ± 0.01 | 0.025 ± 0.01 |
| *lipe* | 1.372 ± 0.12 | 1.021 ± 0.02 | 0.863 ± 0.10 | 0.671 ± 0.10^*^ | 0.819 ± 0.07^*^ | 0.780 ± 0.12 |
| *fasn* | 1.425 ± 0.06 | 0.711 ± 0.06^***^ | 1.191 ± 0.09 | 1.121 ± 0.13 | 0.971 ± 0.07^*^ | 1.700 ± 0.37 |

Data are shown as mean ± SEM (n=5). Significant differences with the control group are indicated by asterisks, determined by one-way ANOVA, followed by Dunnett’s *post-hoc* test. (**p*<0.05, ****p* < 0.001). DM: differentiation medium; GE: genistein; COU: coumestrol; DZN: daidzein; GLY: glycitein; COU: coumestrol; E2: 17β-estradiol.
